# Presidential Symposium: Physiological vs. Molecular Clocks, Studies From Mice to Humans

**DOI:** 10.1093/geroni/igab046.116

**Published:** 2021-12-17

**Authors:** Blanka Rogina

## Abstract

Aging is associated with a functional decline in metabolic, physiological, proliferative, and tissue homeostasis leading to deterioration at the organismal level, and an increased risk for disease and death. Genetic, pharmacological and nutritional interventions have been successfully used to preserve metabolic health, which leads to preserved healthspan and extended longevity. However, the rate at which animals in a population become impaired by age-related frailty and disease is highly variable and several aging clocks that measure different age-modulated processes in the organism are being use as potential markers of the rate of aging. These molecular clocks allow to a more accurate quantification of the biological age of animals. Nevertheless, there is still room for further discussion in terms of the strengths and weaknesses of these biomarkers, in order to probe their biological significance, cellular mechanisms, and epidemiological potential to further explore their long-term benefit of increasing healthspan. This symposium will discuss new approaches to delineate physiological versus molecular clocks based on studies in mice and humans. We will also discuss species-specific metabolic mechanisms based on longitudinal studies in mice, monkeys and humans.

